# PP42 Comprehensive Care Program For Polypharmacy Patients: Cafam Experience (Healthcare Provider Institution – Pharmaceutical Manager)

**DOI:** 10.1017/S0266462325102614

**Published:** 2025-12-29

**Authors:** Jesus Ocana, Carolina Parra, Javier Urrego, Leal Guillermo

## Abstract

**Introduction:**

We live in an increasingly long-lived society with rising rates of multimorbidity, which requires the formulation of multiple medications to treat chronic conditions. This can result in polypharmacy (the use of more than five medications in the same month) with consumption maintained over time, making it necessary for health policies and professionals to jointly resolve this issue to reduce the associated health risks.

**Methods:**

We conducted a before-and-after intervention study in 661 patients over 74 years of age with persistent excessive polypharmacy (≥10 medications for more than six months). The intervention was carried out by the Healthcare Provider Institution primary care team led by a family doctor, accompanied by the pharmacoepidemiology area of the Cafam pharmaceutical manager. After six and 12 months of intervention (a time established to notice the effects of the adjustment), the patients’ medications were evaluated through the pharmacy manager’s dispensing history to identify changes in the pharmacotherapy of prioritized patients.

**Results:**

Some type of adjustment was made to pharmacotherapy for 36 percent of the patients seen in consultation; for 35 percent of the cohort, it was not necessary to modify any aspect of their pharmacological therapy; and for 29 percent, it was not possible to carry out an intervention (users belonged to other cohorts or there was not enough information to define an intervention). Having achieved a 20 percent decrease in use of the group of agents for the treatment of stomach acid disorders during the study period implies progress in terms of reducing future clinical complications and improving pharmacological efficiency.

**Conclusions:**

The intervention of the comprehensive care program led to an improvement in the pharmacotherapy among selected users by reducing the number of prescribed drugs and promoting the rational use of health technologies. In addition, a net reduction of COP67,387,362 million (USD16,846) was achieved in the first year of monitoring the program.

